# Supplementary figures and images for: Two waves of pro-inflammatory factors are released during the influenza A virus (IAV)-driven pulmonary immunopathogenesis
Source: PLoS Pathog. 2020 Feb 26;16(2):e1008334. doi: 10.1371/journal.ppat.1008334 (PMC7062283; doi:10.1371/journal.ppat.1008334)

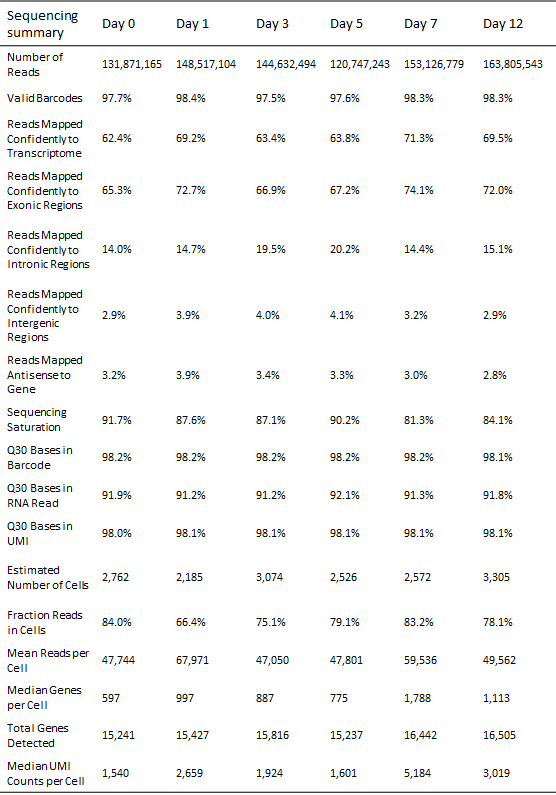

Supplement: S1 Table — (TIF) [file ppat.1008334.s001.tif]

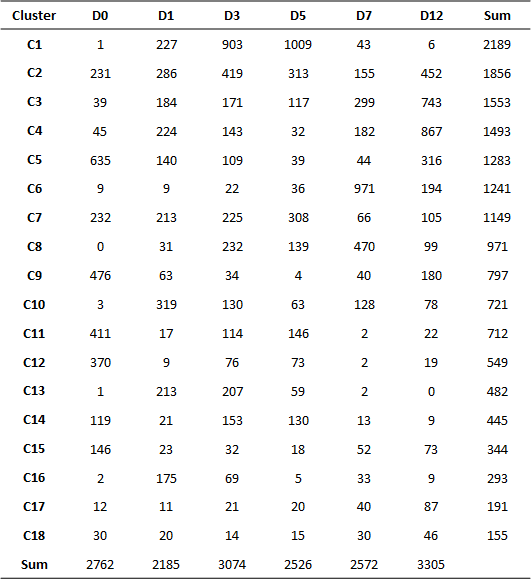

Supplement: S2 Table — (TIF) [file ppat.1008334.s002.tif]

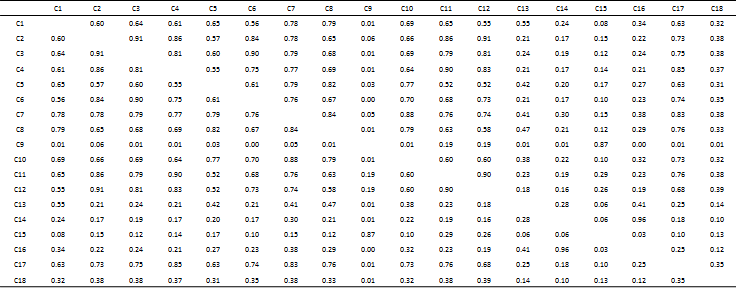

Supplement: S3 Table — (TIF) [file ppat.1008334.s003.tif]

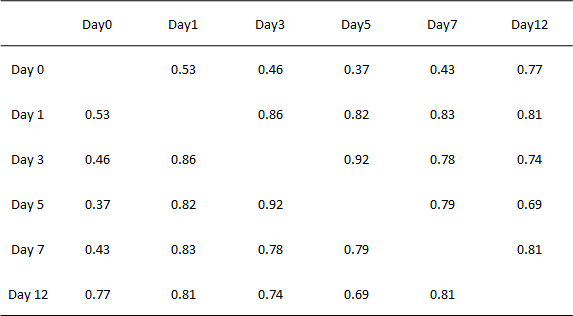

Supplement: S4 Table — (TIF) [file ppat.1008334.s004.tif]

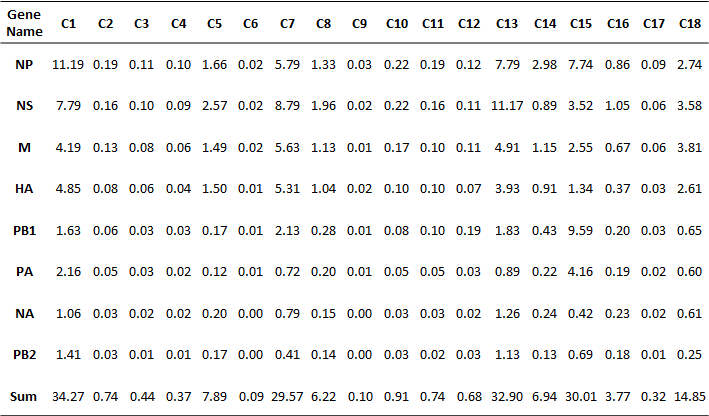

Supplement: S5 Table — (TIF) [file ppat.1008334.s005.tif]

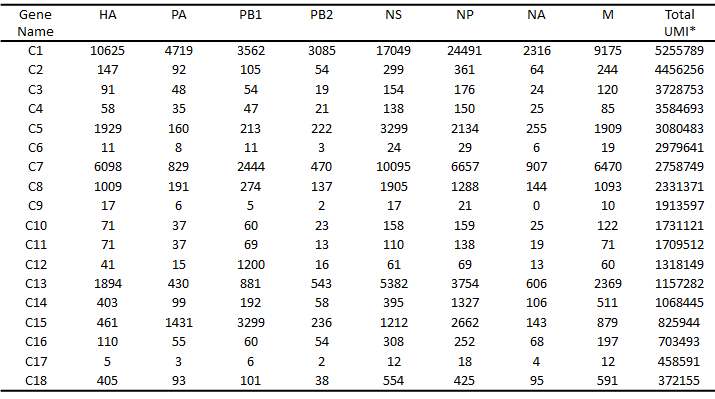

Supplement: S6 Table — (TIF) [file ppat.1008334.s006.tif]

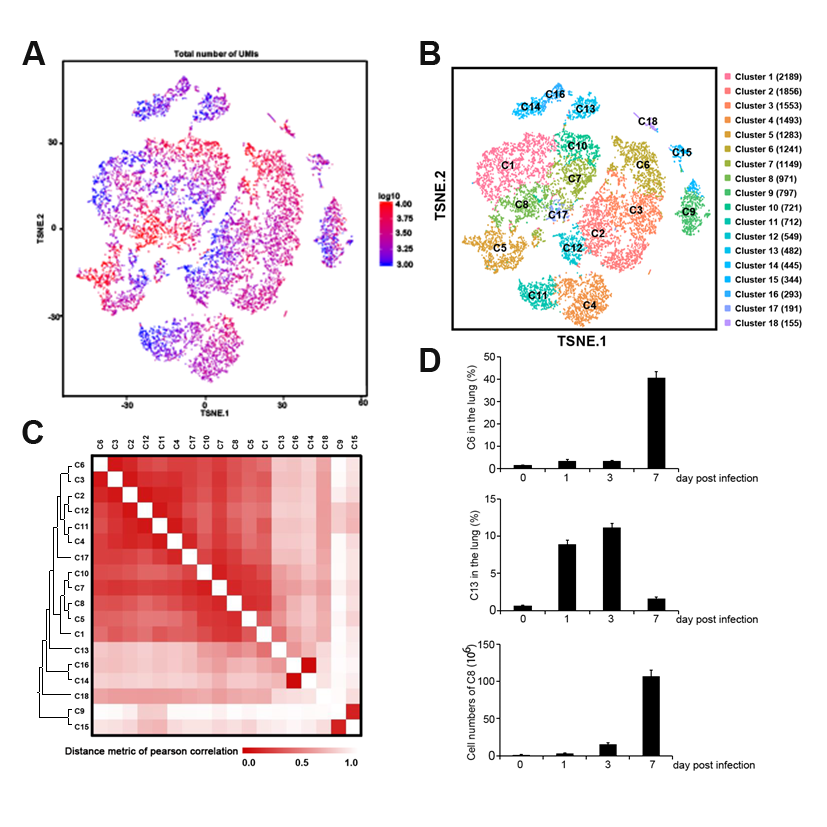

Supplement: S1 Fig — (A) tSNE projection where each cell is colored by log10 of UMI counts. Color scale represents log10 of UMI counts. Each point in the scatter plot represents a cell in the coordinates specified by the two t-SNE components. The color of each point plotted indicates the total number of UMIs for each cell, and these count values are displayed in log10 scale. (B) tSNE maps displaying 16,424 suspended cells from the lung and coloured by the main cell populations based on the unsupervised graph-based, showing the formation of 18 main clusters with the cell numbers in the right panel. (C) Heatmap showing the scaled distances calculated based on pearson correlations for relationships between the normalized mean expression profiles of cells from different clusters. A hierarchical cluster tree constructed based on the distance metric of pearson correlation was shown at the left panel. The numbers represent the percent of all cells from that cluster that are in each day‘s library. (D) Cells of different clusters in the lung were analyzed by FACS analysis at different day post infection. Cells of the lung from mice infected with IAV at the indicated times post infection or from uninfected mice were collected. C6-CD8+ T cells, C8-Pf4+-macrophages, and C13-PD-L1+-neutrophils in the lung were analyzed with FACS analysis, and the cell numbers or frequency were calculated. Data are representative of three independent experiments. (TIF) [file ppat.1008334.s007.tif]

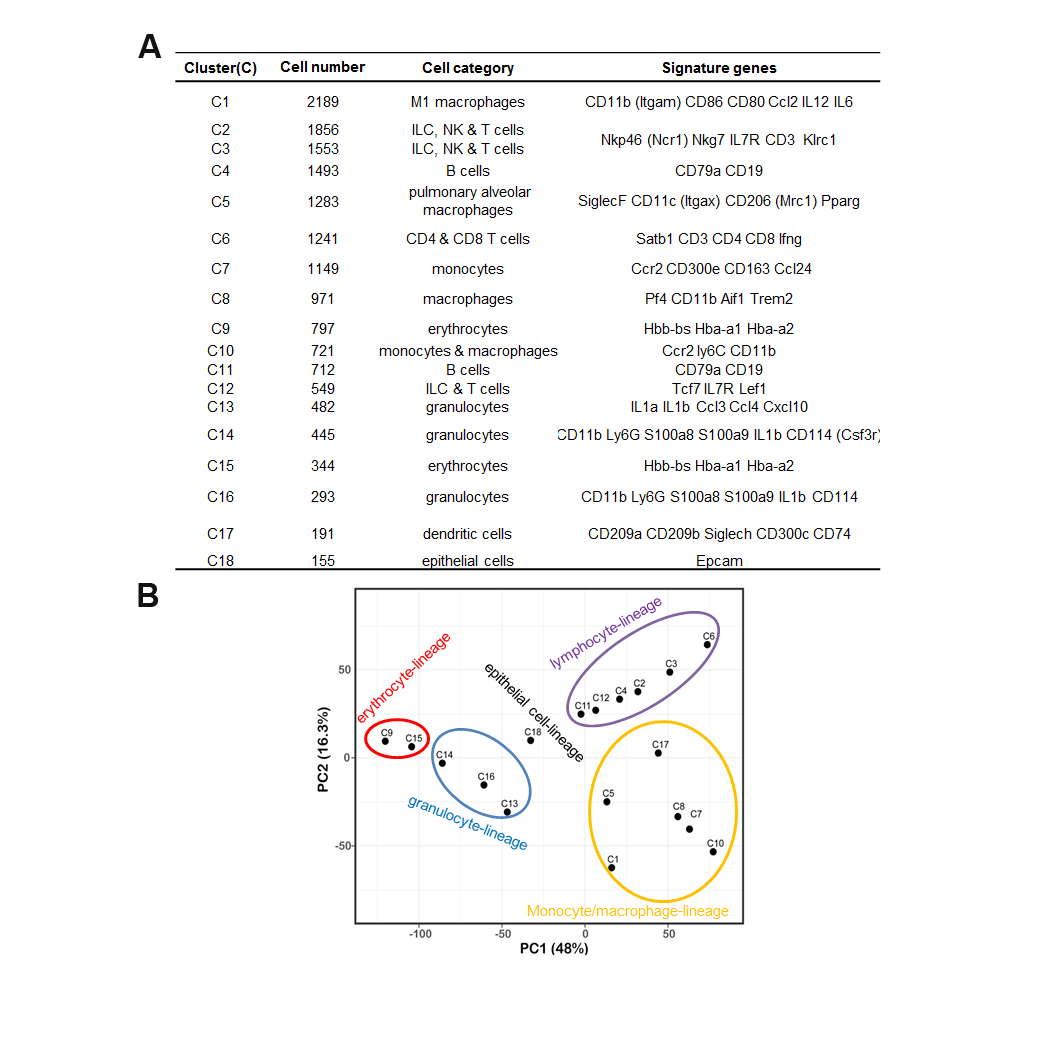

Supplement: S2 Fig — (A) the signature genes of each cluster was shown. (B) PCA analysis of the 18 main clusters Graph-based Clustering. X and Y axis show the principal component 1 and principal component 2 that explain 48% and 16.3% of the total variance, respectively. (TIF) [file ppat.1008334.s008.tif]

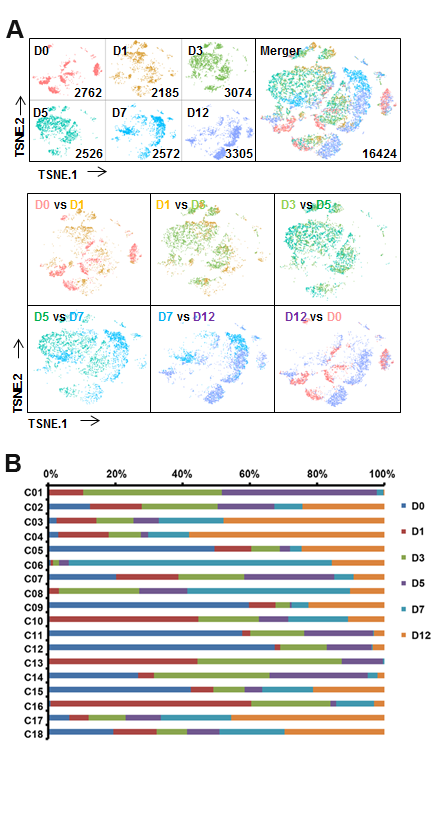

Supplement: S3 Fig — (A) tSNE maps displaying 16,424 cells from the lung of mouse after infected with IAV and colored by the samples of different days post-infection. tSNE maps of different days were combined for comparison with corresponding colors. The data between libraries was normalized by equalizing the read depth between libraries before merging. (B) tSNE maps displaying the comparisons between samples of different days post infection. (C) Proportions of different cell clusters in each library at different days p.i.. (TIF) [file ppat.1008334.s009.tif]

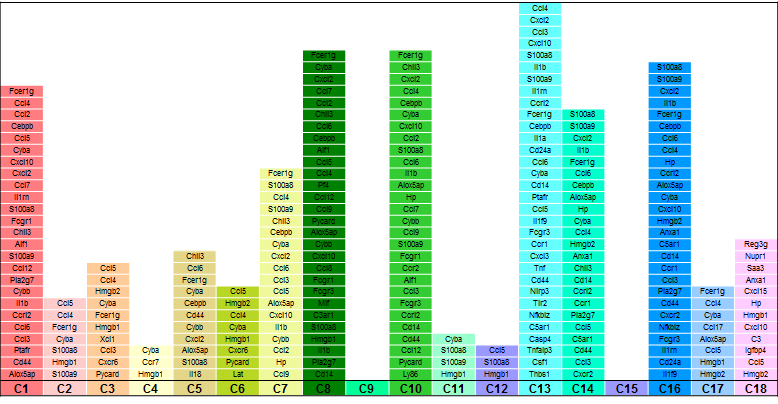

Supplement: S4 Fig — The ranking of gene from top to bottom is based on the mean expression level in each cluster. (TIF) [file ppat.1008334.s010.tif]

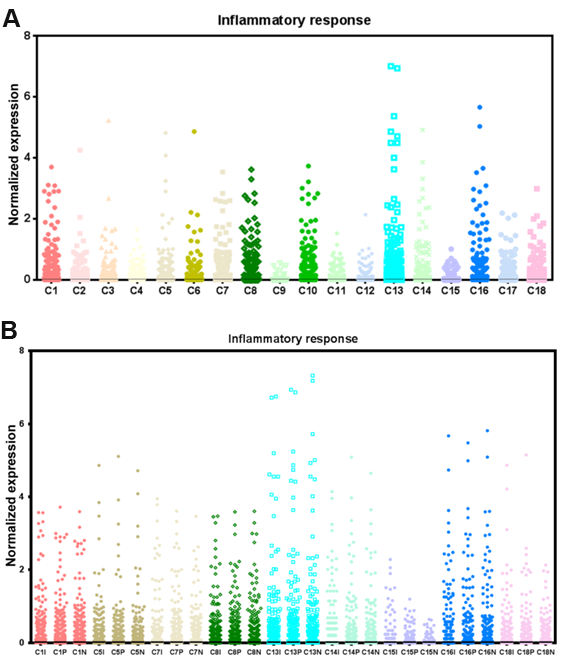

Supplement: S5 Fig — (A) The highlighted clusters with bright color (i.e. C1, C6, C8, C10, C13, C16) were newly emerged and significantly increased post infection. (B) The normalized expression of host 372 genes related to inflammatory response in the significantly infected clusters of different levels of IAV infection (X-axis) (GO: 0006954). The mean expression of each gene was calculated across all cells in the cluster indicated at the Y-axis with log2(x+1) transformed. The cells in the clusters susceptible to IAV infection were divided into highly infected cells (I), potential or lowly infected cells (P), and uninfected cells (N). (TIF) [file ppat.1008334.s011.tif]

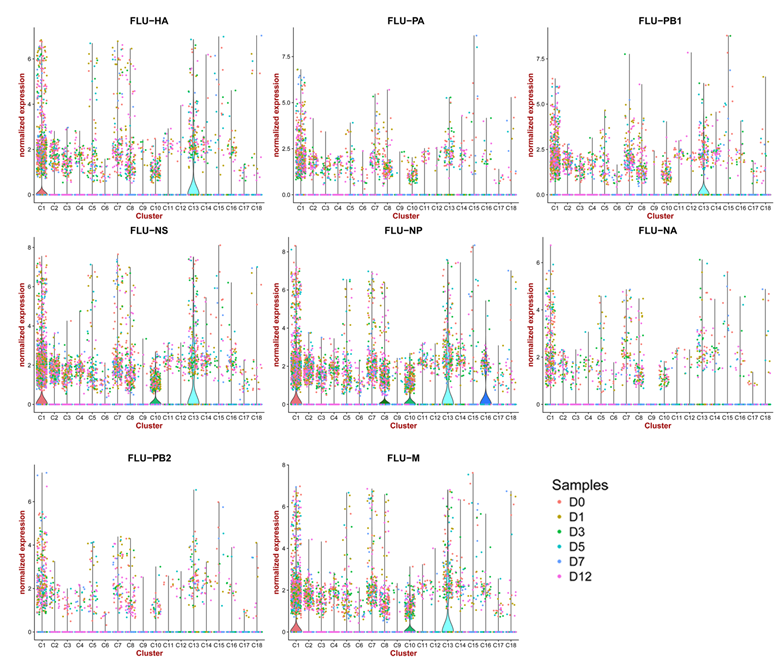

Supplement: S6 Fig — The dots indicate the cells of 18 clusters from different libraries across six time points p.i. with corresponding colors. (TIF) [file ppat.1008334.s012.tif]

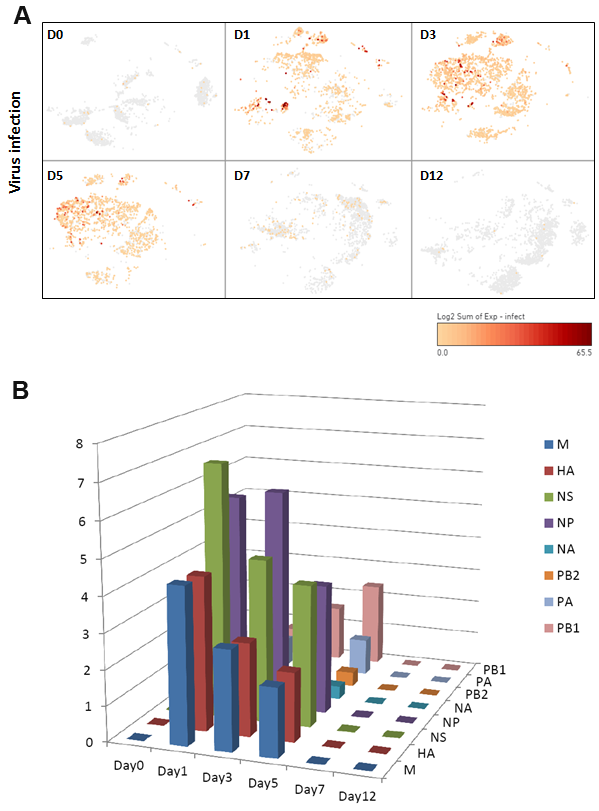

Supplement: S7 Fig — (A) tSNE projection where each cell is colored by log2 sum expression of the eight genes of IAV. (B) The mean expression of IAV eight genes in the single cell libraries from lung at different days post infection. (TIF) [file ppat.1008334.s013.tif]

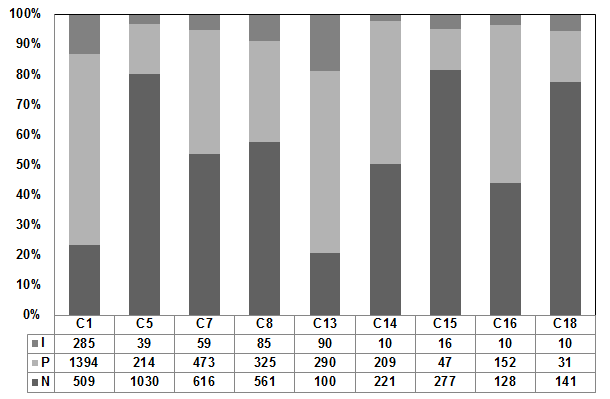

Supplement: S8 Fig — The cells in the clusters susceptible to IAV infection were divided into highly infected cells (I, total UMI counts of viral transcripts ≥8), potential or lowly infected cells (P, total UMI counts of viral transcripts ≥1), and undetected cells (N, UMI counts of viral transcripts = 0). The percentages of highly infected cells (gray), potential or lowly infected cells (light gray), and undetected cells (dark gray) were shown in y axis. The cell counts of different sub-clusters were shown at the bottom. (TIF) [file ppat.1008334.s014.tif]

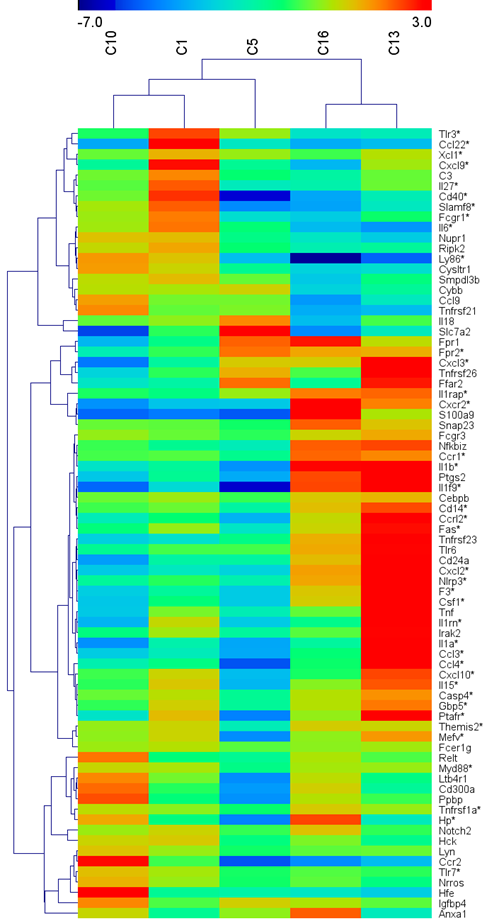

Supplement: S9 Fig — The hierarchical cluster trees were constructed based on the distance metric of pearson correlation among genes or clusters. The asterisk indicates the highly expressed pro-inflammatory genes only at day 1 p.i.. (TIF) [file ppat.1008334.s015.tif]

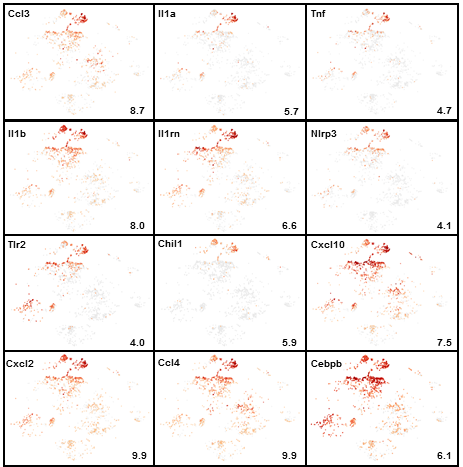

Supplement: S10 Fig — The number at the lower right corner of each box indicates the color up limit for measuring gene expression of single cell. (TIF) [file ppat.1008334.s016.tif]

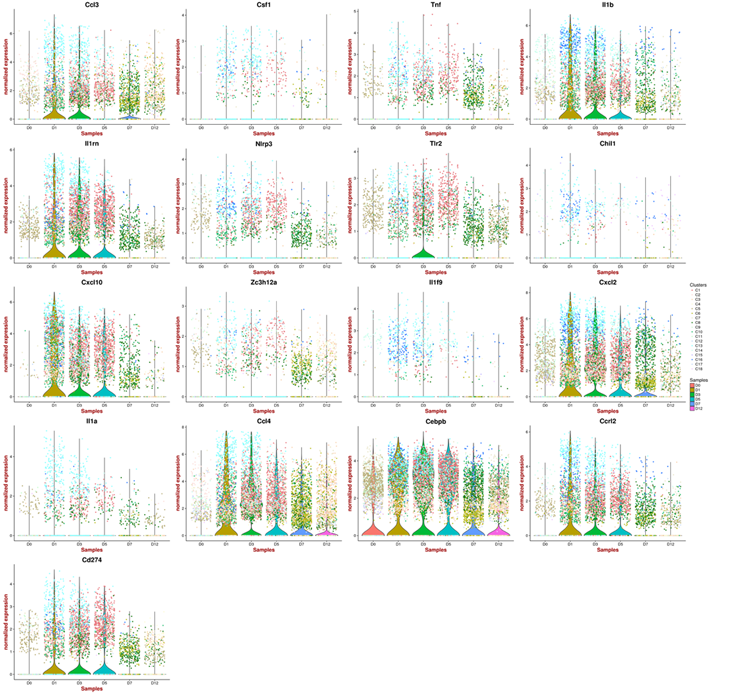

Supplement: S11 Fig — The normalized expression (UMI counts, Y-axis) of the significant genes with high expression related to inflammatory response in the cells of different clusters from different days p.i. (X-axis). The dots indicate the cells from different clusters with corresponding colors. (TIF) [file ppat.1008334.s017.tif]

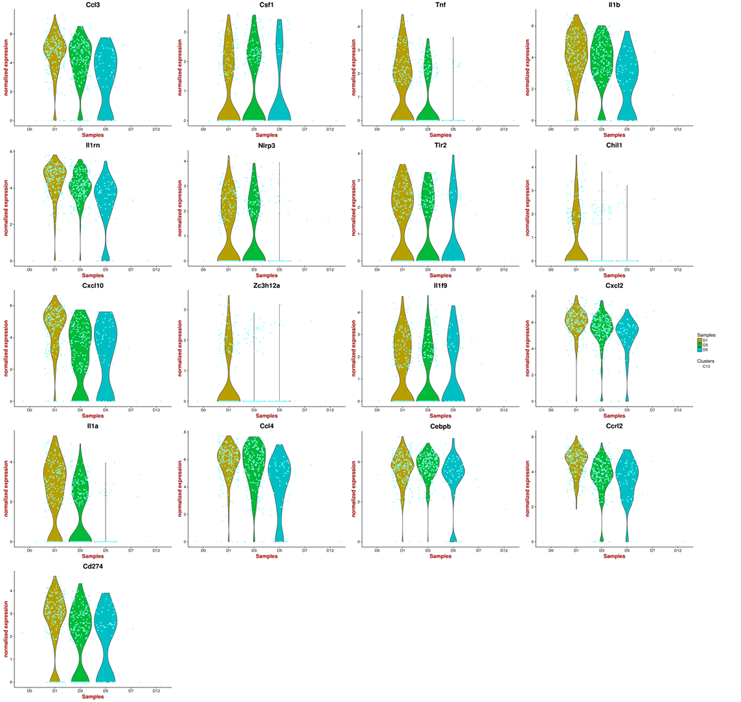

Supplement: S12 Fig — The normalized expression (UMI counts, Y-axis) of the significant genes with high expression at day 1 p.i. related to inflammatory response in the cells from C13 (different days p.i., X-axis). (TIF) [file ppat.1008334.s018.tif]

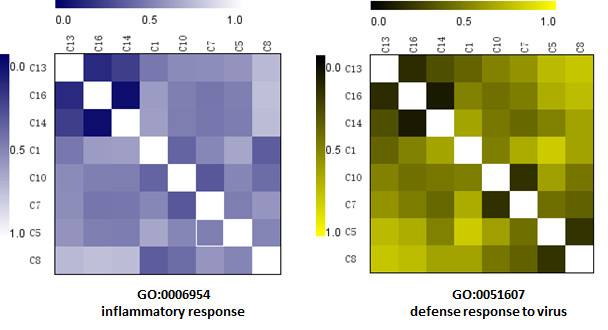

Supplement: S13 Fig — Heatmap showing pearson correlation for relationships between the normalized mean expression profiles of cells from different clusters of myeloid-lineage based on the genes related to GO:0006954 inflammatory response (left) and GO:0051607 defensive response to virus (right). (TIF) [file ppat.1008334.s019.tif]

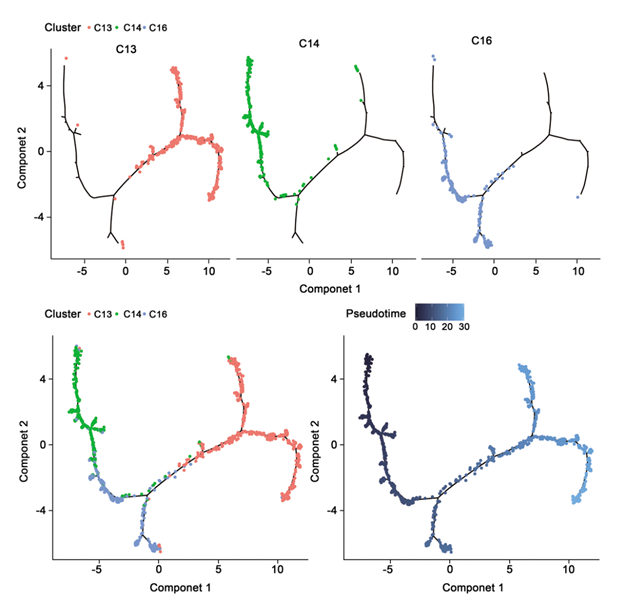

Supplement: S14 Fig — (TIF) [file ppat.1008334.s020.tif]

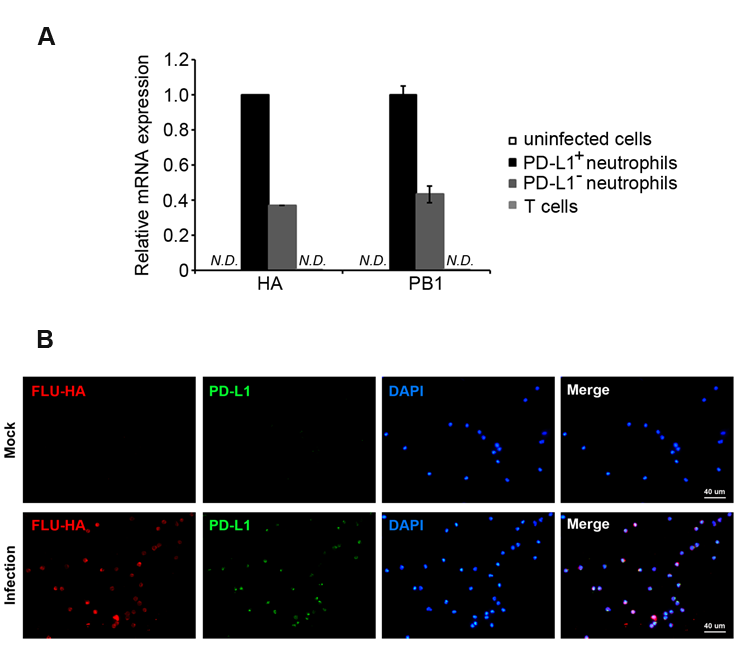

Supplement: S15 Fig — (A) The relative viral mRNA expression in uninfected cells, PD-L1+ neutrophils, PD-L1- neutrophils, and T cells from the lung of mice infected with 0.5 LD50 of influenza A/PR/8/34 (H1N1) viruses was analyzed with qRT-PCR at day 1 p.i.. Data are shown as the means ± SD in one of three independent experiments. N.D. means not detected. (B) Immunofluorescent images showing the neutrophils (CD11b+Ly6G+ cells) isolated from lung of mice uninfected (top panel) or infected (bottom panel) with influenza 0.5 LD50 of A/PR/8/34 (H1N1) viruses at 24 hours p.i. with anti-FLU-HA (red) and anti-PD-L1 (green) antibodies. The nucleus was stained with DAPI (blue). Scale bars, 40 μm. (TIF) [file ppat.1008334.s021.tif]

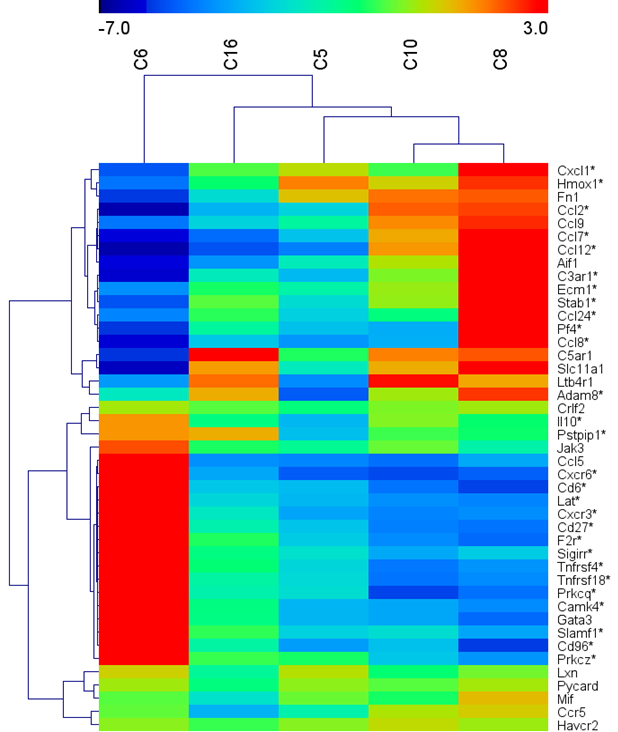

Supplement: S16 Fig — The hierarchical cluster trees were constructed based on the distance metric of pearson correlation among genes or clusters. The asterisk indicates the highly expressed pro-inflammatory genes only at day 7 p.i.. (TIF) [file ppat.1008334.s022.tif]

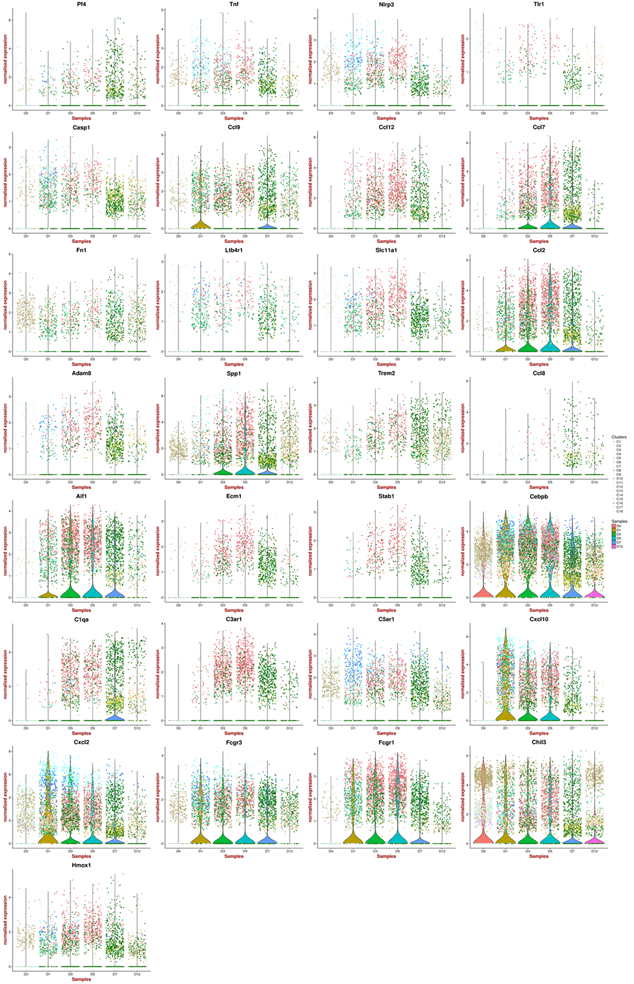

Supplement: S17 Fig — The normalized expression (UMI counts, Y-axis) of the significant pro-inflammatory genes with high expression at day 7 p.i. in the cells of different clusters from different days p.i. (X-axis). The dots indicate the cells from different clusters with corresponding colors. (TIF) [file ppat.1008334.s023.tif]

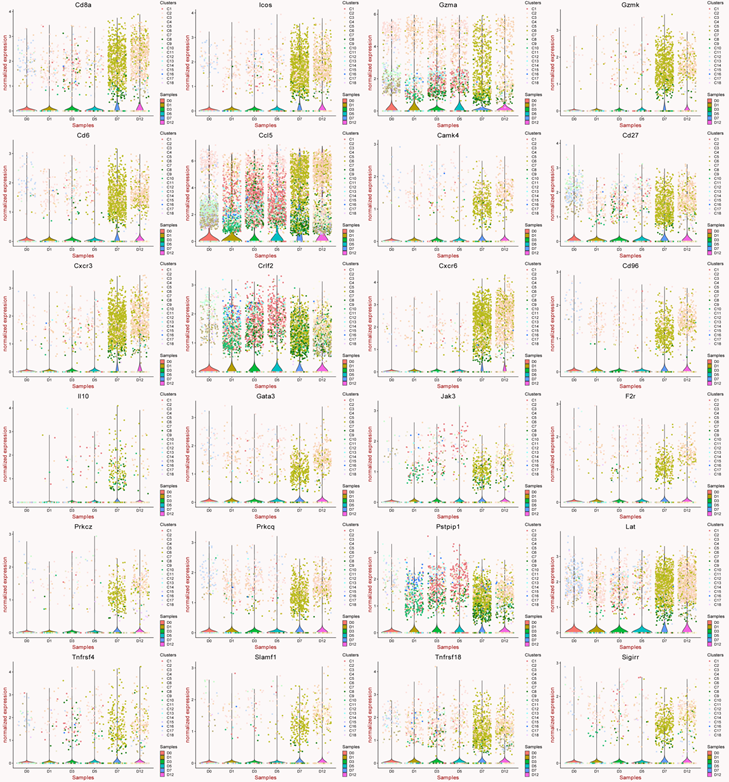

Supplement: S18 Fig — The normalized expression (UMI counts, Y-axis) of the significant pro-inflammatory genes with high expression at day 7 p.i. in the cells of different clusters from different days p.i. (X-axis). These selected genes were mainly significantly high express in C6 cluster at day 7 p.i.. The dots indicate the cells from different clusters with corresponding colors. (TIF) [file ppat.1008334.s024.tif]

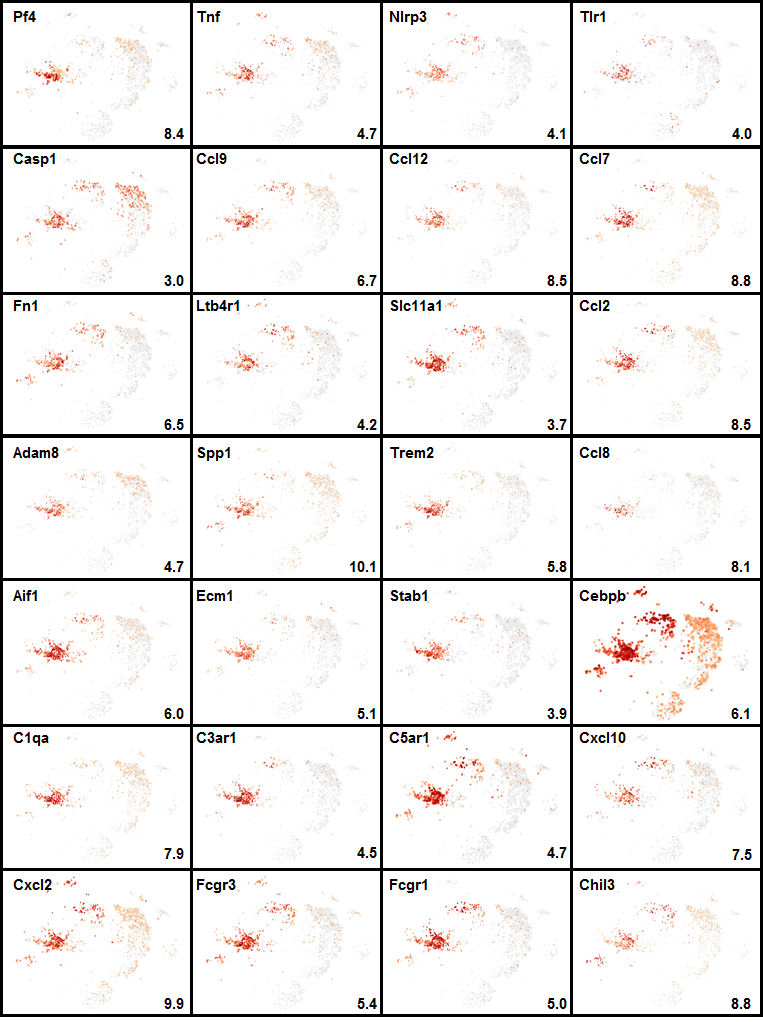

Supplement: S19 Fig — The number at the lower right corner of each box indicates the color up limit for measuring gene expression of single cell. (TIF) [file ppat.1008334.s025.tif]

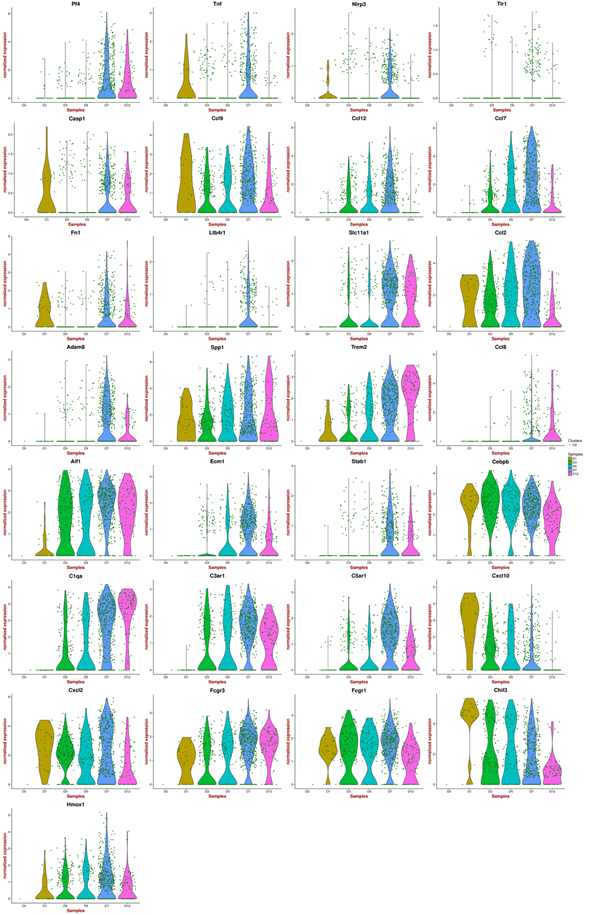

Supplement: S20 Fig — The normalized expression (UMI counts, Y-axis) of the significant pro-inflammatory genes with high expression at day 7 p.i. in the cells from C8 (different days p.i., X-axis). (TIF) [file ppat.1008334.s026.tif]

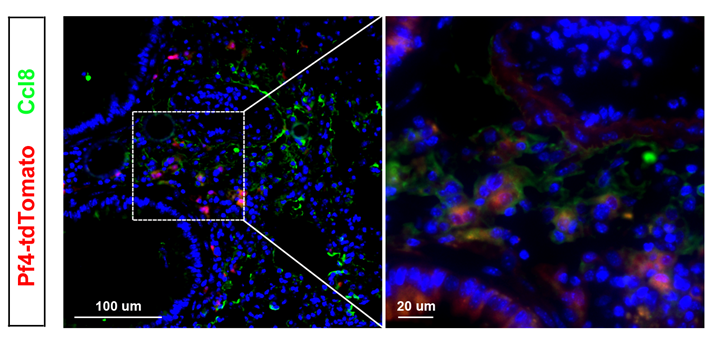

Supplement: S21 Fig — The tdtomato-Pf4 mice were infected with 0.5 LD50 of influenza A/PR/8/34 (H1N1) viruses. At day 7 p.i, cells in the lung were stained with anti-Ccl8 antibodies (green), and the nucleus was stained with DAPI (blue). TdTomato-Pf4 was shown in red. Scale bars, left, 100 μm; right, 20 μm. About 60% of Ccl8+ Pf4-tdTomato+ cells were found within Pf4-tdTomato+ cells. At least 300 cells were scored. (TIF) [file ppat.1008334.s027.tif]

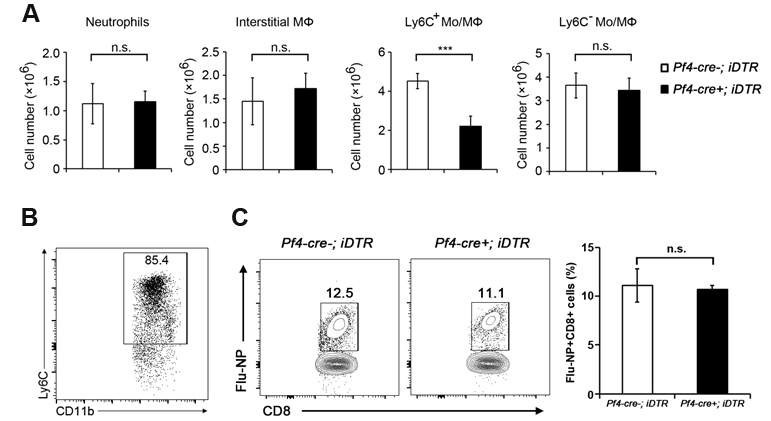

Supplement: S22 Fig — (A) The cell number of neutrophils (CD11b+Ly6G+), interstitial macrophages (CD11b+CD11c+CD64highMHCII+CD24-), and mature monocytes and macrophages (CD11b+MHCIIintCD24-Ly6C+/Ly6C-) was analyzed in Pf4-cre-; iDTR mice and Pf4-cre+; iDTR mice. (B) Flow cytometry of Pf4+ cells from the lung sample of Pf4-tdTomato mice infected with IAV. The cells were gated on Pf4-tdTomato+CD11b+. Number in quadrants indicates percent Pf4-tdTomato+CD11b+Ly6C+ cells. (C) Left panel: Flow cytometry of infected CD8+ T cells (Flu-NP+CD8+) of lung samples from Pf4-cre-; iDTR mice and Pf4-cre+; iDTR mice. Numbers in quadrants indicate percent infected CD8+ T cells. Right panel: Frequency of Flu-NP+CD8+ of the lung samples in Left. Data are shown as the means ± SD in one of three independent experiments. ***, P < 0.001 (Student t test, n = 5). n.s. means not significant. (TIF) [file ppat.1008334.s028.tif]

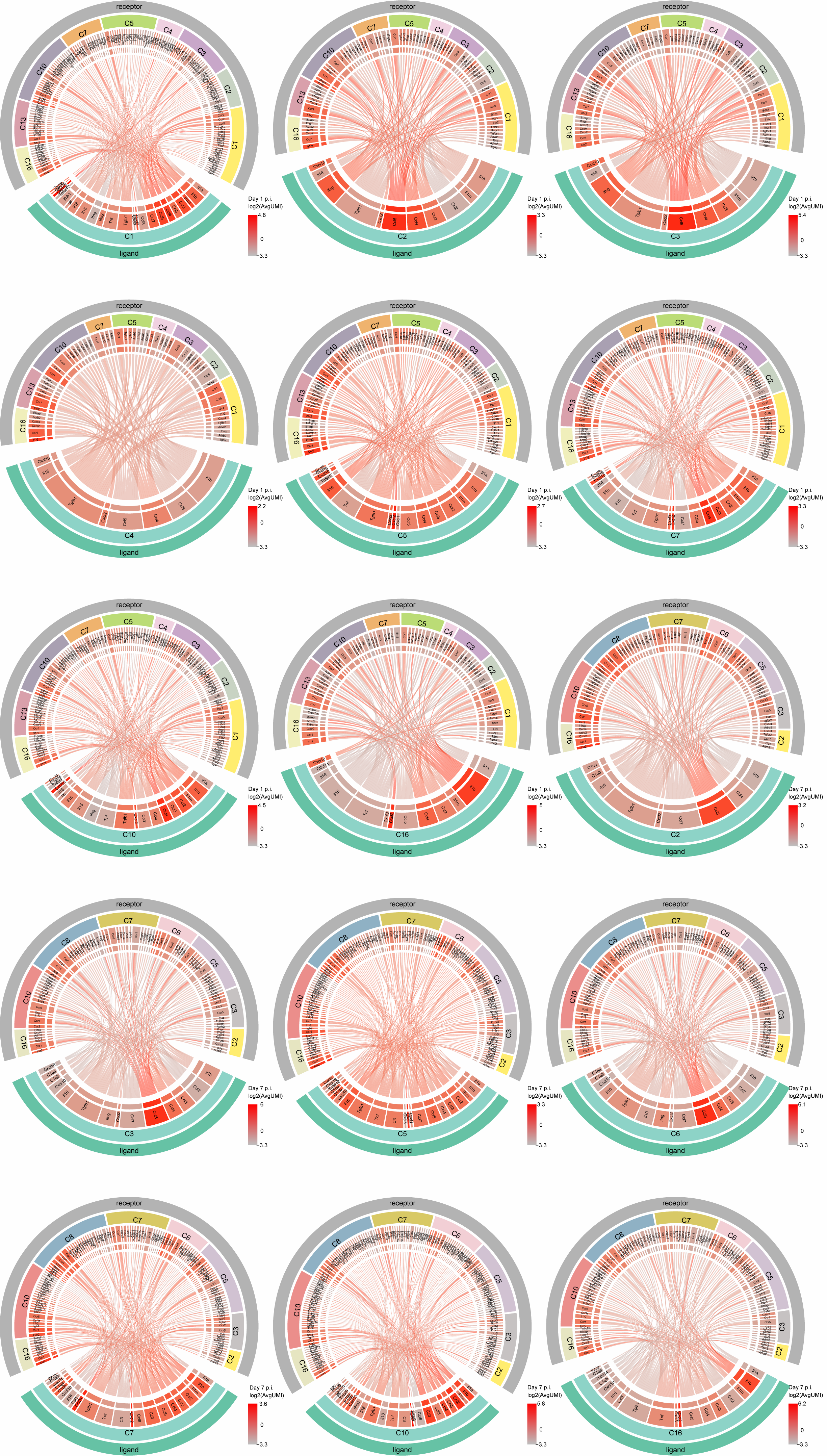

Supplement: S23 Fig — Line connections indicate the literature supported ligand and receptor interactions. The average UMI counts of all LR genes were log2 transformed, and highlighted with the gradual red color in the graph according the transcription level of LR genes. The LR interaction lines were colored in accordance with the transcription level of ligand or receptor genes in the main cluster. (TIF) [file ppat.1008334.s029.tif]

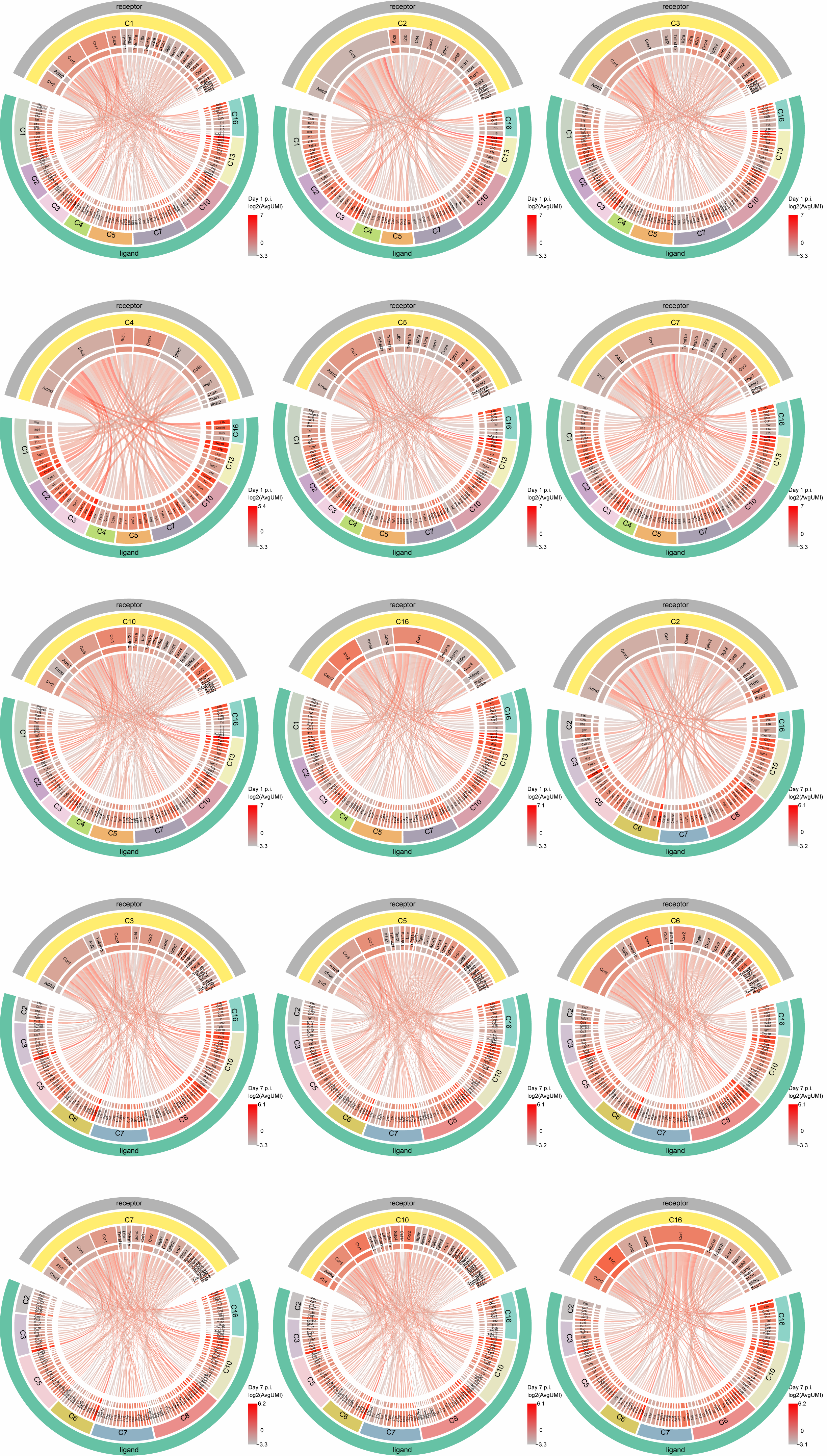

Supplement: S24 Fig — Line connections indicate the literature supported ligand and receptor interactions. The average UMI counts of all LR genes were log2 transformed, and highlighted with the gradual red color in the graph according the transcription level of LR genes. The LR interaction lines were colored in accordance with the transcription level of ligand or receptor genes in the main cluster. (TIF) [file ppat.1008334.s030.tif]
