# Supplementary material for: Two waves of pro-inflammatory factors are released during the influenza A virus (IAV)-driven pulmonary immunopathogenesis
Source: PLoS Pathog. 2020 Feb 26;16(2):e1008334. doi: 10.1371/journal.ppat.1008334 (PMC7062283; doi:10.1371/journal.ppat.1008334)
Supplement: Attachment — Submitted filename: response letter.docx [file ppat.1008334.s031.docx]

We thank for the constructive suggestions from reviewers, which have helped improve our manuscript a lot. Please see our answers to the concerns, point by point:

*Responses to Questions*

***Part I – Summary***

*Please use this section to discuss strengths/weaknesses of study, novelty/significance, general execution and scholarship.*

*Reviewer #1: The authors present a very interesting study examining the inflammatory response in different cell types over time.*

*One major weakness is the n=3 mice per groups and number of cells examined (sometimes less than 100)*

We thank this reviewer for his/her comments saying that our study is very interesting. Also, we are sincerely sorry for not using more mice in the study. However, mouse model is the most conventional model in study of influenza virus infection, and the same condition of IAV infection shows very small intragroup diversity in mice of IAV infection. We feel that n=3 mice per groups is enough for our study though it is better for us to use more mice per groups in the study.

The subtypes of immune cells in lung of influenza A virus (IAV)-infected mice are complex, and the infection of IAV will induce the amplification and migration of immune cells. Thus, some cell types were actually of low proportion in lung of mice, such as C16 in day 0 and C11 in day 7. Though with low proportion, we were still able to identify them as a distinct cell cluster according to their significantly expressed genes. Based on the low proportion of some cell types, it is technically unachievable to guarantee for all the cell types more than 100. However, since we utilized scRNA-seq in our study, one hundred cells theoretically represent one hundred replicates of one type of cells.

*Reviewer #2: The manuscript by Zhang and colleagues details a single cell RNASeq study of mouse lung before and following influenza infection over 5 time points. In general, the presentation of the vast data set is beautifully executed with cutting edge bioinformatic approaches. The authors find numerous interesting data including that viral load in cells did not impact inflammatory cytokine production, that early inflammatory gene expression is mostly from Pdl1+ neutrophils, and that later inflammatory gene expression tracks with pf4+ macrophages. The authors follow up on the pf4+ macrophage finding using a DTR system to delete these cells and assess inflammation endpoints. Interactome data at the end of the study reveal several autocrine loops that drive inflammation. While the concept that neutrophils drive early and macrophages late inflammation in response to influenza infection is not new, the resolution of the kinetic data presented is novel and very interesting to the field. The study could be improved by follow up regarding the* *Pdl1+ PMN and addition of data regarding interferon producing cells.*

We thank this reviewer for his/her comments saying that we found numerous interesting data, and the resolution of the kinetic data presented is novel and very interesting to the field. Regarding the concerns on Pdl1+ PMN and addition of data regarding interferon producing cells, we will address in details in the following answers.

*Reviewer #3: Understanding of the dynamics and coordination of the immune response to influenza A virus infection is important for biomarker discovery and identification of targets for intervention. This study uses the murine influenza model and single-cell RNA sequencing to analyse the changes in pulmonary cellular composition over the first 12 days of infection. At day 0, 1, 3, 5, 7 and 12 post-infection, lung cell suspensions from 4 mice were mixed and analysed by 10x Chromium. At each time-point ~2000-3300 individual cells underwent scRNA-seq. By tSNE, cells were clustered and clusters categorised into cell type by their expression of signature genes. A Gene Ontogeny module was used to probe each cluster at every time-point to identify those clusters with high expression of inflammatory response genes. One cluster was associated with early inflammatory response at day 1 and this was found to be PD-L1 expressing neutrophils; while another cluster associated with inflammatory response at day 7 was found to be PF4 (or CXCL4) expressing macrophages. Additionally, some correlation analysis was performed to identify associations between chemokine/cytokine receptors and their ligands expressed in different clusters.*

*Overall, the manuscript is confusingly written, with the rationale for each analysis incompletely expressed and many essential details overlooked. In many cases, supplementary data are cited but not explained in the Results. Some experiments are not adequately explained so interpretation is often difficult. The study is basically descriptive but the authors claim to identify mechanisms of pathogenesis. There are generally issues with the English and the paper would benefit from a thorough review of the language.*

We are sincerely sorry for poor written and inappropriate expression. We have carefully reviewed the language thoroughly.

***Part II – Major Issues: Key Experiments Required for Acceptance***

*Please use this section to detail the key new experiments or modifications of existing experiments that should be absolutely required to validate study conclusions.*

*Generally, there should be no more than 3 such required experiments or major modifications for a "Major Revision" recommendation. If more than 3 experiments are necessary to validate the study conclusions, then you are encouraged to recommend "Reject".*

*Reviewer #1: The authors have examined groups of 3 mice at multiple timepoints. This is relatively low and may not represent variability. In addition, different types of cells were analysed however for some groups less than 100 cells were examined. This cell numbers are very low. It was not clear what the authors have done to account for this?*

As discussed above, mouse model is the most conventional model in study of influenza virus infection, and the same condition of IAV infection shows very small intragroup diversity in mice of IAV infection. We feel that n=3 mice per groups is enough for our study though it is better for us to use more mice per groups in the study.

The subtypes of immune cells in lung of influenza A virus (IAV)-infected mice are complex, and the infection of IAV will induce the amplification and migration of immune cells. Thus, some cell types were actually of low proportion in lung of mice, such as C16 in day 0 and C11 in day 7. Though with low proportion, we could still identify them as a distinct cell cluster according to their significantly expressed genes. Based on the low proportion of some cell types, it is technically unachievable to guarantee for all the cell types more than 100. However, since we utilized scRNA-seq in our study, one hundred cells theoretically represent one hundred replicates of one type of cells.

*A t test was used throughout the manuscript. This is not appropriate when comparing multiple groups.*

A two-tail student *t* test was used in Fig 2H, Fig 3D, Fig 4B, Fig 4C, Fig 4D, Fig 4G, and Fig 4H, all of which are comparing between two groups. Thus, we believed that the student *t* test analysis was appropriately used in the study. The missing statistics labels have been added in the revised manuscript (Fig 2H and Fig 4B in the revised manuscript).

*Some of the figures are very small and axis labels are hard to read eg Fig 3B*

We have enlarged the labels as possible to make them easily to read (Fig 1D, Fig 1E, Fig 2A, Fig 2E, and Fig 3B in the revised manuscript).

*Reviewer #2: 1) Mechanistic Studies – The authors conducted mechanistic studies to confirm the role of pf4+ macrophages. Did depletion of these cells impact PMN of other macrophage populations? How specific was the depletion method utilized for pf4+ cells alone (beyond the data showing no impact on T cells). The authors should target pdl1+ PMN early during infection to generate similar data implicating these cells as critical inflammatory drivers.*

According to the reviewer’s suggestion, after the depletion of Pf4^+^ macrophages in *Pf4-cre****^+^****; iDTR* mice, we found that only Pf4^+^ macrophages were decreased, while the cell numbers of PMN and other macrophage populations were not altered (Fig S21A in the revised manuscript). Of note, Pf4^+^ macrophages belong to CD11b^+^Ly6C^+^ macrophages (Fig S21B in the revised manuscript). These results indicated that the method used to delete Pf4^+^ cells is specific enough for us to study the function of Pf4^+^ macrophages.

In agree with the reviewer’s opinion, we also think that it is interesting to study the role of PD-L1^+^ PMN during early infection. However, it is technically difficult to utilize the diphtheria toxin induced system in the absence of *Pd-l1*-cre mice. In addition, Pf4 is specifically expressed in C8 (Fig 3B), while PD-L1 are broadly expressed in different cell subsets, including C1, C5, C8, C10, C13, C14, C16, and C17 (Fig 2E). Thus, even though C13 expressed the highest PD-L1 among all the clusters, other cells expressed PD-L1 would also be eliminated within the *Pd-l1*-diphtheria toxin induced system, during which it will be difficult for us to analyze the specific function of PD-L1^+^ PMN in lung of IAV infection.

*2) IFN Producing Cells – As it is known that type I, II, and III IFNs regulate inflammation during influenza infection, it would be helpful to highlight data showing which cells make these antiviral cytokines during the infection time course.*

We have re-analyzed the expression of type I, II, and III IFNs in all the cell subtypes during IAV infection (Figure 1 for the reviewers). We found the expression of IFNG was high in C3 (ILC, NK & T cells) and C6 (T cells) and various types of interferon receptors were highly expressed in most immune cells. Most of I and III IFNs were reported highly expressed in epithelial cells. However, as only a small fraction of epithelial cells were collected in our study, the expression of I and III IFNs in these cells was difficult for analyzing.

Figure 1 for the reviewers. The normalized expression (UMI counts) of I, II, and III IFNs in the lung in different clusters.

*3) Tissue Dissociation – The authors observed almost no epithelial cells in this study. They should comment on how the single cell suspension methodology appears to have selected for leukocytes and how this limits interpretation in the discussion.*

As the reviewer mentioned, low level of C18-epithelial cells was found (about 1% in total cells of lung) in our study since the lung was homogenized using lung dissociation kit rather than enzymatic digestion. Epithelial cells have strong intercellular adhesion, which would be removed after homogenized and filtered through a 70 μm nylon mesh filter, especially for the highly infected epithelial cells which undergo apoptosis. However, immune cells were infiltrated into alveoli, which can be separated easily and then made into single cells suspension by cell filter. In our study, we aimed to analyze the immune cells in lung isolated from different time points during IAV infection, and provide an atlas of immune response in lung during IAV infection. Thus, we only focused on the immune cells. The lung epithelial cells did show great interaction with immune cells infiltrated in lung. However, we believed that the immune responses among immune cells have shown many insights of the pulmonary immune processes during IAV infection. The limitation of this method has been discussed in the revised manuscript (Line 236-243).

*Reviewer #3: Line 163. The GO term 0006954 was chosen to identify clusters making an inflammatory response. This is a very biased approach. How was this GO term chosen over the many others? The authors should justify this strategy and discuss its limitations. Could a more unbiased strategy be used?*

To avoid the bias of choosing the genes representing in inflammatory response, we have searched the Gene Ontology databases comprehensively, and found that the GO term 0006954 is the best match in inflammatory response containing the most inflammatory response genes reported compared with other terms. The official annotation of this term in the Gene Ontology database is “inflammatory response” (https://www.ebi.ac.uk/QuickGO/term/GO:0006954), the child terms of which are the direct descendants of various biological process involved in inflammatory response. Based on the supporting evidence from the official database, this strategy is comprehensive and objective, avoiding the arbitrariance of artificial judgment. In addition, to further confirm the detail ability of inflammatory response in the cell clusters, we have already listed the expression values in single cell of the representative genes with the experimental verification in the figures.

*Line 169. In this analysis, the epithelial cell cluster C18 has a much lower proportion of IAV transcript-expressing cells than monocyte/macrophage and granulocyte clusters. Can this be correct? Productive IAV replication in vivo is unlikely in monocytes and neutrophils, while epithelial cells are the main target for IAV. How can this be explained? Could there be a technical reason for this analysis to be skewed? More explanation of Figures S6, S7 and Table S6 is necessary.*

As discussed above, low level of C18-epithelial cells was found (about 1% in total cells of lung) in our study since the lung was homogenized using lung dissociation kit rather than enzymatic digestion. Epithelial cells have strong intercellular adhesion, which would be removed after homogenized and filtered through a 70 μm nylon mesh filter, especially for the highly infected epithelial cells which undergo apoptosis. Besides, different subtypes of influenza viruses show different cell tropism at different stage of infection (Richard W. Compans, Michael B. A. Oldstone (Ed.). Current Topics in Microbiology and Immunology, Volume 145, Page 19-20). IAV infection initiates in the respiratory tract and spreads in the lung. However, the viruses do not spread into the whole lung tissue based on the dose used in a previous study (Pan W, et al., Nat Comm. 2013; 4:2369). Thus, it is difficult for most of the epithelial cells in lung infected with IAV *in vivo*. In addition to the low proportion of epithelial cells, it resulted in that the epithelial cell cluster C18 has a much lower proportion of IAV transcript-expressing cells than monocyte/macrophage and granulocyte clusters.

More descriptions for Figures S6, S7 and Table S6 have been added in the revised manuscript (Line 168-180).

*Line 172. This is unexpected. Surely more viral components ought to trigger greater PRR signalling? Again, could there a technical reason for the expression counts of IAV transcripts not to be correct?*

Some previous studies have demonstrated that the cells under both extracellular exposure and intracellular infection can exhibit a significant response in addition to a significant bystander response (Ablasser A, et al., Nature. 2013; 503:530-4; Nguyen TA et al. Mol Immunol. 2017; 86:16-22). The antiviral responses of immune cells are not just activated through auto-signals including PRR signaling. They may be activated by two different ways including auto-signal from the virus-infected cells or the signals from intercellular communications. The auto-signal like PRR signaling exited in some cell types such as C15 that harbored high virus RNA and showed high antiviral response. In the meantime, no visible correlation between viral load and host response level was observed in some cell types such as C13. Therefore, we thought that the phenomenon described in Fig S5 could be caused by cell-cell interaction and communication *in vivo*. The uninfected or lowly infected immune cells could be activated by cytokines or chemokines from other immune cells and generate antiviral factors or other pro-inflammatory factors.

The expression counts analyses were performed by following the official method which was also adopted by many other researches (<https://www.10xgenomics.com/resources/publications>). For the Gene Expression Algorithms Overview, please see <https://support.10xgenomics.com/single-cell-gene-expression/software/pipelines/latest/algorithms/overview>. Altogether, we believed that the phenomenon we found is technically correct and the interpretation is reasonable. The similar phenomenon has also been observed in a previous report (Cohen M et al., Cell Syst. 2018;6:679-691.e4).

*Line 310. “high level of virus infection in C13-neutrophils was revealed” This should be validated. Could viral transcripts be quantified from sorted cells by qPCR?*

Actually, we have validated this result with qRT-PCR and immunofluorescence assays which indicated that high mRNA and protein level of virus HA was detected in C13-neutrophils (Fig 2H and Fig S9 in the previous manuscript, or Fig 2H and Fig S15B in the revised manuscript). For further validation, we sorted out C13-neutrophils and total neutrophils for qRT-PCR to detect the mRNA expression of virus HA and PB1, and found that C13-neutrophils harbored higher level of virus transcripts than total neutrophils. Uninfected cells and T cells were taken as negative controls in the experiments (Fig S15A in the revised manuscript).

*Line 233. Does depleting Pf4-expressing cells only affect this population of macrophages? What effect does it have on CXCL4-induced chemotaxis, if any? The cited reference is regarding megakaryotes. More data on the model in the context of the lung is required to support the authors' interpretation.*

As discussed above, we found that only Pf4^+^ macrophages were decreased, while the cell numbers of PMN and other macrophage populations were not altered after the depletion of Pf4^+^ macrophages in *Pf4-cre****^+^****; iDTR* mice. Of note, Pf4^+^ macrophages belong to CD11b^+^MHCII^int^CD24^-^Ly6C^-^ macrophages. These result indicated that the method used to delete Pf4^+^ cells is specific enough for us to study the function of Pf4^+^ macrophages (Fig S21A and S21B in the revised manuscript). CXCR3 is most abundant receptor for CXCL4, and mainly expresses on CD8^+^ T cells (Figure 2 for the reviewers). As the population of CD8^+^ T lymphocytes was unaffected after the depletion of C8 Pf4-positive macrophages, we thought that CXCL4-induced chemotaxis would not be affected after the depletion of C8.

The cited report in the manuscript regarding megakaryotes indicated that Pf4 (CXCL4) is the marker for megakaryotes in lung. However, we found that the C8 Pf4-positive cells in our study were much closer to C5/C1/C17-macrophage-lineage in the correlation analysis (Fig 3F) and PCA analysis (Fig 3H), but not megakaryotes as reported. The C8 Pf4-positive cells secreted a large number of cytokines as macrophages did (Fig 4), which further confirmed our conclusion. It is notable that Pf4 can be taken as a marker of macrophages (Calaminus SD, et al., PLoS One. 2012;7:e51361). Moreover, we found that Pf4^+^ cells from lung of mice infected with IAV at 8 p.i. highly expressed CD11b, which is the marker of macrophage (Figure 3 for the reviewers). Thus, we suggested that the C8 Pf4-positive cells discovered in the lung of mice at the late stage of IAV infection in our study were different from megakaryotes which mainly exist in the bone marrow of mice.

Figure 2 for the reviewers. The normalized expression (UMI counts) of Pf4 receptor Cxcr3 in different clusters was analyzed.

Figure 3 for the reviewers. Flow cytometry of CD11b expression of Pf4^+^ cells cells from the lung samples collected at day 8 p.i. from B6 mice.

***Part III – Minor Issues: Editorial and Data Presentation Modifications***

*Please use this section for editorial suggestions as well as relatively minor modifications of existing data that would enhance clarity.*

*Reviewer #1: Can the dose of virus given to the mice be represented as PFU to allow comparison to other studies.*

0.5 LD_50_ of PR8 per mice is equal to 50 PFU of PR8 per mice. It has been added in the methods part of the revised manuscript (Line 448-449).

*Reviewer #2: 1) Supp Figures – The description of the data in the numerous Supp Figures is sparse or not present. Particularly for Supp Fig8-12 and 15-18, these are glossed over in the results section.*

We thank the reviewer for pointing out the problem. More explanation of the Figures mentioned by the reviewer has been added in the revised manuscript (Line 203-211; Line 253-261).

*2) Grammar – There are some minor grammatical issues in the text.*

Sorry for these mistakes. We have carefully reviewed the language thoroughly.

*Reviewer #3: Line 144. Clusters were categorised according to “known markers of major cell types”. How were these chosen and validated?*

According to previous reports (Cohen et al., Cell. 2018;175:1031-1044.e18; Schelker M et al. Nat Comm. 2017; 8(1):2032), we chose the classical markers as the definition of immune cell subtypes. For example, the CD3, CD4, and CD8 genes were taken as the basis for the definition of T cell cluster (Fig S2).

*Line 152. “The complex intrinsic components of cells…” What does this mean?*

Sorry for the misleading, we have changed it to “The heterogenous components of immune cells” in the revised manuscript (Line 153).

*Line 165. The phrase “exerted strong pro-inflammatory response” is not an accurate reflection of what the method shows; this is a transcriptional signature, not a functional readout. Suggest re-phrasing.*

We have changed it to “generated many pro-inflammatory factors” in the revised manuscript (Line 166-167).

*Line 173. “indicated the roles of innate immune cells in controlling IAV-driven pneumonia” The analysis has not shown the roles only described the presence and changing composition of immune cells. Sugges re-phrase.*

We have changed it to “described the composition of immune cells during IAV-driven pneumonia” in the revised manuscript (Line 191).

*Line 186. Supplementary figures have not been explained.*

More explanation of the Figures mentioned by the reviewer has been added in the revised manuscript (Line 168-186; Line 203-211; Line 253-261).

*Line 187-9. The correlation analysis shows correlation between several clusters that have been labelled by the authors as granulocyte-like but does not actually show granulocyte characteristics as claimed.*

We agree with the reviewer and have changed it to “granulocyte-like” in our manuscript (Line 213).

*Line 191. Where did the data from eosinophils and basophils come from?*

The transcriptional profile data of eosinophils and basophils were retrieved from the NIH SRA database with the accession code SRP040656 (<https://www.ncbi.nlm.nih.gov/sra/>). We have explained it in the revised manuscript (Line 216-217).

*Line 192. “further confirmed by” The pseudotime ordering analysis suggests but does not confirm the transition between clusters. Suggest re-phrase.*

We have changed it to “further demonstrated by” in the revised manuscript (Line 219).

*Line 201 & Figure 2H. What does “much higher” mean? Where are the statistical tests shown?*

Sorry for the mistake. The missing statistical labels have been added in the figure of the revised manuscript.

*Line 203. What does “super-activated” and “cytokine-hypersecreted” mean and in relation to what? Also, only cytokine transcripts are shown, not protein. Suggest re-phrase.*

Sorry for the inappropriate expression. As discussed above, we have validated this result with qRT-PCR and immunofluorescence assays which indicated that high mRNA and protein level of virus HA was detected in C13-neutrophils. Thus, we have changed this sentence to “The isolated PD-L1^+^ neutrophils (PD-L1^+^CD11b^+^Ly6G^+^) at day 2 p.i. harbored much higher viral RNA and IL-1α mRNA levels, as well as higher Ccl3, Ccl4, IL-1β mRNA levels, compared with PD-L1^-^ neutrophils (PD-L1^-^CD11b^+^Ly6G^+^), indicating that the C13 PD-L1^+^ neutrophils generated high pro-inflammatory cytokine mRNA level in the lung at early stage of IAV infection” in the revised manuscript (Line 225-230).

*Line 204. Only viral transcripts are shown, no “virus protein HA”. Suggest re-phrase.*

Sorry for the mistake. As discussed above, we have already analyzed the virus protein HA in the study. However, we located the supplemental figure in the incorrect position. We have re-located it to the right position in the revised manuscript (Fig S15B in the revised manuscript).

*Line 227. Error in citing figures: 3F is a correlation analysis, 3G is the PCA analysis and there is no GO enrichment analysis shown.*

Sorry for these mistakes, we have corrected them in the revised manuscript (Line 269).

*Line 241. It is unclear how the experiments in Figure 4A and 4B were performed. The figure legend says that RT-PCR was done in Pf4+-macrophages but these are ablated in the Pf4-cre+ mice. There is nothing about ELISA in the materials and methods, and it is unclear what sample type CCL8 and CCL2 have been measured in.*

Sorry for these mistakes. The qRT-PCR was performed in the whole immune cells of lung. The figure legend has been corrected in the revised manuscript (Line 855). The CCL8 and CCL2 were measured in BALs of mice (Line 859). The method for ELISA has been added in the materials and methods of the revised manuscript (Line 495-499).

*Line 255. “indicated that Pf4-positive macrophages were the precursors of AMs” I do not think the data show this. Reduction in AMs could be indirect. Suggest re-phrase*

We have changed it to “indicated that Pf4-positive macrophages may be the precursors of AMs” in the revised manuscript (Line 302).

*Line 285. “further confirmed the interaction” I do not think the data actually confirms these interactions since it only shows correlations.*

We have changed it to “correlation” in the revised manuscript (Line 332).

*Line 304. “play a leading role” The authors have shown these cells expressing inflammation-related genes at day 1 but not actually demonstrated their role.*

We have changed it to “are the major contributor to” in the revised manuscript (Line 351-352).

*Line 370. “demonstrated many new mechanisms” I do not think any new mechanisms have been demonstrated. Sequential waves of innate and adaptive immune cells have been shown in many respiratory viral infection systems. Although neutrophils are thought of as anti-bacterial, they have also been implicated in antiviral responses. I think the authors are over-claiming.*

We have changed it to “demonstrated many new phenomena” in the revised manuscript (Line 417).

*Materials and methods. The method for ELISA should be described.*

The method for ELISA has been added in the methods part of the revised manuscript (Line 495-499).

*Figure 2A: The symbols # and ## are too small to see.*

We have enlarged the labels as possible to make them easily to read.

*Figure 2A legend: “# indicated the highly-expressed genes” Do # and ## indicate statistically significant differentially expressed genes? Suggest re-phrasing legend to clarify.*

As suggested, we have re-phrased the legend. The right rows marked with # indicated the highly-expressed genes (Log2 fold change ≥1) at day 1 p.i.. The right rows marked with two ## indicated the highly-expressed genes (Log2 fold change ≥1) at day 7 p.i. (Line 800-802).

*Figure 2B legend: “normalized expression” Normalised how and to what?*

We used the z-score normalization method to normalize the UMI counts of the single-cell transcripts. The heatmap showed the normalized expression (Z-score) of the pro-inflammatory genes in various cells of clusters with high ratios at day 1 p.i.

*Figure 2C legend: “were unregulated” What does this mean?*

Sorry for the spelling mistake. It should have been “upregulated”. We have corrected it in the revised manuscript and have carefully reviewed the language thoroughly (Line 312; Line 808).

*Figure S5 legend. “The highlighted clusters…” There are no highlighted clusters. Later in the legend, there is repetition of a sentence.*

We thank the reviewer for pointing out the problem. The highlighted clusters (i.e. C1, C6, C8, C10, C13, C16) were labeled with the bright color and further explained in the revised legend. The repetition of some sentences was deleted in the revised legend.

*Figure S7. This and plots like it (e.g. S17) are almost uninterpretable. There are too many colours that are poorly explained and the superimposed dots make it impossible to appreciate the time-line.*

In the revised manuscript, we added more interpretation of these figures and colors. To present the time-line, the colors of superimposed dots indicating the days post-infection have been labeled and explained.

*Figure S10. How have these pro-inflammatory genes here (other representative genes in other figures e.g. S11, S12, S16) been selected?*

The pro-inflammatory genes selected and exampled here were the significant upregulated genes (log2 fold change >1) which are important for illustrating that the PD-L1^+^ neutrophils are the major contributor to the first wave of pro-inflammatory factors during the IAV infection.

*Figure S18. Please provide statistical test results to show significant changes over time.*

The statistics test results for the significant changes over time has been shown in the Supplementary Excel table of the revised manuscript.

*Figure S19. This is not very convincing. Is there any quantitative co-expression analysis available?*

The quantitative co-expression analysis has been added in the figure legend of Figure S20 in the revised manuscript.

*Figure S20. Please provide a plot summarising frequencies between groups and statistics.*

The frequency and statistics between groups has been shown in the revised manuscript (Fig S21C in the revised manuscript).

Again, we thank all the reviewers for their constructive comments.
