# Supplementary material for: Two waves of pro-inflammatory factors are released during the influenza A virus (IAV)-driven pulmonary immunopathogenesis
Source: PLoS Pathog. 2020 Feb 26;16(2):e1008334. doi: 10.1371/journal.ppat.1008334 (PMC7062283; doi:10.1371/journal.ppat.1008334)
Supplement: Attachment — Submitted filename: response letter.docx [file ppat.1008334.s032.docx]

We thank again for the constructive suggestions from reviewers. Please see our answers to the concerns, point by point:

*Reviewer's Responses to Questions*

*Part I - Summary*

*Please use this section to discuss strengths/weaknesses of study, novelty/significance, general execution and scholarship.*

*Reviewer #1: The authors have not addressed my concern of n=3 for the mouse studies. Could major findings be validated with additional experiments?*

Thanks for the suggestion from the reviewer. We also believed that the major findings should be validated with experiments, and we actually did several experiments in the original figures to validate our scRNA-seq data including Figure 2F-2G validating that PD-L1^+^ neutrophils were the major contributors for the first pro-inflammatory factors at day 1 p.i., and Figure 3C-3D and Figure 4 validating that PF4^+^ macrophages were the major contributors for the second pro-inflammatory factors at day 7 p.i.. Besides, more experiments to validate the scRNA-seq data have been performed in the figure 1 for the reviewer. In agreement with scRNA-seq data, we found that C6-CD8^+^ T cells and C8-Pf4^+^-macrophages mainly occurred at day 7 post infection, while C13-PD-L1^+^-neutrophils mainly occurred after day 1 post infection, indicating that the scRNA-seq data is repeatable.

**Figure 1 for the reviewers. Cell numbers of different cell types in the lung were analyzed by FACS analysis at different day post infection.** (A) The percentage of C6-CD8^+^ T cells in the lung; (B) The percentage of of C8-Pf4^+^-macrophages in the lung; (C) Cell numbers of C13-PD-L1^+^-neutrophils in the lung.

*Reviewer #2: The authors have carefully addressed my concerns and I think the utility of the data for the field is high.*

We thank for comment of this reviewer.

*Reviewer #3: Overall, the manuscript has improved substantially. It certainly presents a large body of work and has sought to validate the transcriptomics findings with a variety of follow-up studies. The findings are interesting and the dataset represents a useful resource. The flow and readability of the paper are much better, although there still remain some grammatical errors here and there.*

We thank this reviewer for his/her comments saying that our manuscript has improved substantially, and that we validated the transcriptomics findings with a variety of follow-up studies. We have carefully analyzed these errors the reviewer mentioned below. The point by point details have been addressed in the following answers.

*Part II – Major Issues: Key Experiments Required for Acceptance*

*Please use this section to detail the key new experiments or modifications of existing experiments that should be absolutely required to validate study conclusions.*

*Generally, there should be no more than 3 such required experiments or major modifications for a "Major Revision" recommendation. If more than 3 experiments are necessary to validate the study conclusions, then you are encouraged to recommend "Reject".*

*Reviewer #1: (No Response)*

*Reviewer #2: (No Response)*

*Reviewer #3: Line 249: the authors mention and dismiss the C6 cluster as a source of pro-inflammatory cytokine expression. Looking at their data, these are lymphocytes and likely to represent CD8+ T cells, which would be expected to be expanded and recruited at the day 7 timepoint. Ideally, this population would be analysed in more detail as their contribution is not negligible. The authors may feel that this is beyond the scope of the paper, given that they have focused solely on innate cells, but additional analysis of this population would provide a more complete view and increase the impact. The importance of other subsets is further supported by the lack of complete abrogation following depletion of the Pf4+ macrophages (Fig 4C-D).*

We thank the reviewer for this valuable suggestion. According to the reviewer’s suggestion, we have analyzed the C6 cluster in our revised manuscript. The C6 cluster is mainly composed of activated CD8^+^ Cells highly expressing the inflammatory factors including the lymphocytic activation molecules and the genes involved in the protective cytotoxic T cell response. The expression results of these specific genes in C6 cluster were shown in Figure 1C, Figure 3A and supplementary Figure S16-S18 in the revised manuscript.

*Part III – Minor Issues: Editorial and Data Presentation Modifications*

*Please use this section for editorial suggestions as well as relatively minor modifications of existing data that would enhance clarity.*

*Reviewer #1: (No Response)*

*Reviewer #2: (No Response)*

*Reviewer #3: Line 56: this implies that pandemics are yearly. Suggest rephrase.*

We have deleted “each year” of the sentence in the revised manuscript.

*Line 61: "we characteristically monitored..." The meaning of this is unclear. Suggest rephrase.*

We have deleted “characteristically” of the sentence in the revised manuscript.

*Line 63: "resources for the release..." The meaning of this is unclear. Suggest rephrase.*

We have changed it to “contributors” in the revised manuscript.

*Lines 121 "high-quality" and 127 "the most stable and accurate". Suggest removing this qualifiers as they are disputable.*

We have removed the qualifiers in the revised manuscript according the the reviewer’s suggestion.

*Line 143 "Some significant genes..." This remains unclear. Do the authors mean that all genes meeting their statistical criteria are shown or that only a selected subset, and if the latter, what were the selection criteria?*

Not all the genes that meet the statistical criteria (Log2 fold change >1, *P*-value <0.01, Benjamini-Hochberg adjusted) were shown. We only selected genes that well represent major cell types according to previous study (Cohen M, Giladi A et al., Lung Single-Cell Signaling Interaction Map Reveals Basophil Role in Macrophage Imprinting. Cell. 2018 Nov 1;175(4):1031-1044.e18; Schelker M, Feau S et al., Estimation of immune cell content in tumour tissue using single-cell RNA-seq data. Nat Commun. 2017 Dec 11;8(1):2032). For instance, Gzmb is not the markers for CD8^+^ T cells. However, it is well known that Gzmb is highly expressed in CD8^+^ T cells as well as ILC and NK. The specific markers together with these selected genes are more accurate to represent a single cell type.

*Line 187-189. From the response to reviewers, the authors suggest that this lack of correlation is due to inflammatory activation by cytokines and other bystander effects. It would be worth discussing this in more detail in the Discussion.*

More discussion of this issue has been added in the revised manuscript.

*Figure 4G is still extremely difficult to interpret. Suggest a plot showing data from the C8 cluster alone.*

According to the reviewer’s suggestion, we have showed data from the C8 cluster alone in Figure 4G in the revised manuscript.

Again, we thank all the reviewers for their constructive comments.
