# Supplementary material for: Two waves of pro-inflammatory factors are released during the influenza A virus (IAV)-driven pulmonary immunopathogenesis
Source: PLoS Pathog. 2020 Feb 26;16(2):e1008334. doi: 10.1371/journal.ppat.1008334 (PMC7062283; doi:10.1371/journal.ppat.1008334)
Supplement: Attachment — Submitted filename: response letter.docx [file ppat.1008334.s033.docx]

We thank again for the constructive suggestions from reviewers. Please see our answers to the concerns, point by point:

*The manuscript is much improved and the added data through these revisions have greatly improved the value of these experiments to the field. This is a very interesting and provocative study.*

We thank for the suggestion for all the reviewers to help us improve the manuscript.

*In the response to Reviewer #1's issue regarding the number of samples analyzed and respond in the response to review but did not add the data shown in "Figure 1" in response to the review to the actual manuscript that generally addresses the issue raised by the reviewer. They should include it as supplementary data with more information on how these experiments were performed.*

According to the reviewer’s suggestion, we have added the data in the Fig S1D in the revised manuscript, and more details of the experiment have been added in the figure legend of Fig S1D in the supplementary materials of the revised manuscript.

Again, we thank all the reviewers for their constructive comments.
